# The contribution of LM to the neuroscience of movement vision

**DOI:** 10.3389/fnint.2015.00006

**Published:** 2015-02-17

**Authors:** Josef Zihl, Charles A. Heywood

**Affiliations:** ^1^Department of Psychology, Max Planck Institute of Psychiatry, Ludwig Maximilian UniversityMunich, Germany; ^2^Department of Psychology, Science Laboratories, Durham UniversityDurham, UK

**Keywords:** movement vision, akinetopsia, cerebral motion blindness, patient LM

## Abstract

The significance of early and sporadic reports in the 19th century of impairments of motion vision following brain damage was largely unrecognized. In the absence of satisfactory post-mortem evidence, impairments were interpreted as the consequence of a more general disturbance resulting from brain damage, the location and extent of which was unknown. Moreover, evidence that movement constituted a special visual perception and may be selectively *spared* was similarly dismissed. Such skepticism derived from a reluctance to acknowledge that the neural substrates of visual perception may not be confined to primary visual cortex. This view did not persist. First, it was realized that visual movement perception does not depend simply on the analysis of spatial displacements and temporal intervals, but represents a specific visual movement sensation. Second persuasive evidence for functional specialization in extrastriate cortex, and notably the discovery of cortical area V5/MT, suggested a separate region specialized for motion processing. Shortly thereafter the remarkable case of patient LM was published, providing compelling evidence for a selective and specific loss of movement vision. The case is reviewed here, along with an assessment of its contribution to visual neuroscience.

## Introduction

“In 1983, the world of neurology witnessed two surprises. The first was the publication of a paper by Zihl et al. describing a patient who has lost the ability to see objects in motion following a bilateral cerebral vascular lesion in cortex outside the striate area. … It was the first description of cerebral motion blindness. … The second surprise was that, although a single case study, it was immediately accepted by the neurological and, more generally, by the neurobiological world, without a murmur of dissent.” With this statement, Zeki introduced his review article on visual motion blindness, for which he coined the term “cerebral akinetopsia” (Zeki, 1991, p. 811). Moreover, he contrasted the silent acceptance of the report of akinetopsic patient LM with the fate of earlier reports of cases of cortical color blindness, so-called cerebral achromatopsia, which were met with some dissent (Zeki, 1990). Undoubtedly, the publication of this exceptional single case, along with its positive acceptance by the neuroscientific community, has stimulated research on movement vision in the fields of psychology and neurobiology. This does not mean that before 1983 knowledge about movement vision was sparse, and evidence of specific processing of visual motion signals in the brain was insubstantial. But the unique case of LM provided the final “missing link” between evidence based on experiments on principles of movement vision in normal observers on the one hand, and the neuroanatomical and neurophysiological evidence of how the brain deals with visual motion information on the other. As will be mentioned below, there was rich indirect evidence for a separate representation of movement vision in the visual brain before LM indicating, together with other evidence of functional segregation in the primate visual cortex (Zeki, 1978), that the visual brain is functionally specialized. What was missing was unequivocal evidence that movement vision can be *specifically* and *selectively* disturbed after acquired brain injury, a fact that was predicted on the basis of the psychological and neurobiological evidence that already existed. Early reports suggesting functional specialization of the visual brain had, in general, aroused considerable controversy (see Zeki, 1993, for a comprehensive review). In his comprehensive monograph on visual disturbances after occipital damage, Poppelreuter (1917/1990) pleaded for such a concept, but also stressed the paucity of evidence: “These few examples, put together rather loosely, might be sufficient for the present to demonstrate our aim of not tolerating the neglect of pathological disorders of all these separate functions merely because they co-occur with the ‘geometrical’ facts of lost portions of the visual field” (p. 21). In 1983, the time seemed ripe for the neuroscientific community to accept and integrate a report of cerebral motion blindness as final confirmatory evidence for the individual representation of movement vision in the brain.

## Evidence for a particular status of movement vision in the visual modality before LM

In his comprehensive paper on visual motion perception, Brown (1931) evaluated the state of research at his time by summarizing that “in the last half century of psychological investigation few specific problems of perception have elicited so many researches and have been the basis for so much theoretical controversy as the visual perception of movement. The reason for this is not far to seek. From the earliest laboratory studies to the most recent it has become increasingly clear that the inconstant correlation between the physical events in the stimulus and the phenomenal events in the perception of movement could not be explained by the ordinary psychophysical concepts. … In all the work that has been done, no investigation has concerned itself primarily with the functional characteristics of the perception of velocity. Various investigators … have observed lack of correlation between the velocity of the stimulating movement and the phenomenal velocity” (pp. 199–200). This theoretical and conceptual dilemma in psychophysics stimulated Brown to investigate the effects of various stimulus variables, e.g., observer distance, size of moving field, degree of homogeneity of the surround of the moving field, component elements in the moving field, size and orientation of the moving stimulus, direction of movement, field brightness and afferent vs. efferent movement perception. The main outcome of his experimental work was that “velocity is perceived directly …. The visual perception of velocity follows dynamic laws that are not immediately deducible from the velocity of the stimulus as physically defined” (Brown, 1931, p. 231). Twenty-three years later Gibson (1954), reviewing the state of the art on the then available empirical evidence on “how do we see motion” and its implications, concluded that “there is plenty of evidence that *visual motion* is a ‘sensory’ variable of experience. It has a kind of intensity (speed) and a kind of quality (direction). … But more than any sensory impression, it *fails to correspond to the physical stimulus presumed for it*. Whatever the stimulus for motion might be, it is *not* simply motion in the retinal image. … It cannot be assumed, that a movement is the same thing in the object, the retina, the brain, and consciousness” (p. 310–311). Interestingly, as early as 1881 Exner had similarly argued that visual movement perception does not depend on the (independent) analysis of spatial displacements and temporal intervals, but represents a specific visual movement sensation (Exner, 1888).

The search for further particular characteristics of movement vision was picked up again by Carlson (1962) and Sekuler and Ganz (1963). These authors presented more direct psychophysical evidence for velocity and direction sensitivity of the human visual motion system using a selective adaptation paradigm, which can be understood as a specific transient functional inhibition (Weisstein, 1969). Subsequent research on human movement vision was inspired by the then exciting neurophysiological evidence of direction- and velocity-dependent analysis of stimulus motion in the extrastriate visual cortex of the cat (Hubel and Wiesel, 1962; Baumgartner et al., 1964) and based on the observation, reported by Barlow and Hill (1963) in the rabbit and by Hubel and Wiesel (1965) in the cat, of the diminution of neuronal responses with repeated stimulation with the same motion stimuli. Pantle and Sekuler (1968), Sekuler et al. (1968) and Pantle (1970) used the same paradigm and found further empirical evidence for velocity- and direction-sensitive visual analysis in human observers. Using a similar psychophysical paradigm, Ritter et al. (1973) replicated and extended the findings of selective and specific adaptation effects of stimulus velocity and movement direction. The interocular transfer of these effects supported the hypothesis of a central site of stimulus analysis, i.e., where visual neurons receive inputs from both eyes (e.g., Hamilton and Lund, 1970; Raymond, 1993). These findings were mainly interpreted in the context of what was then known about primate visual cortical neurophysiology. Wurtz (1969) found a category of neurons in the striate cortex of alert, fixating monkeys which “were rapidly adapting and responded most vigorously to a moving stimulus” (p. 741) and also exhibited directional selectivity; the most vigorous response was found for stimulus velocities of 8–12°/s. The fundamental question, where in the visual cortex visual motion analysis is performed, was finally answered by Zeki (1974) who reported apparent specialization for visual motion in an extrastriate cortical area in the posterior bank of the temporal sulcus in the rhesus monkey (area V5). This area receives a direct and highly convergent input from striate cortex and its neurons are motion selective and also chiefly directionally selective. This important finding was later confirmed by many other authors and led, together with evidence of cortical mechanisms of processing of other visual stimulus attributes, to the concept of functional specialization in the visual cortex (Zeki, 1978). As defined later by positron emission tomography, the visual cortical area in question in humans is at the boundary of Brodmann areas 19 and 37 at the temporo-parieto-occipital pit (Zeki et al., 1991). Irrespective of the question of whether V5/MT represents the “candidate” area in the extrastriate visual cortex, based on the psychophysical, neurophysiological and also neuroanatomical evidence available before 1983, one would have predicted that injury to a particular extrastriate cortical structure should result in a selective and specific impairment of movement vision. A major obstacle to this expectation had already been formulated by the neuroanatomist in Campbell (1905, p. 145): “It is almost impossible for nature to restrict a damaging lesion to the cortex, and to the cortex only, in question.” Injury to the visual cortex usually causes more than one visual dysfunction. Visual movement perception may, therefore, be secondarily impaired, e.g., because of bilateral homonymous visual field defects or impaired visual contrast sensitivity, or because of other pathological conditions, for example, reduced visual acuity (Wood and Kulikowski, 1978), amblyopia (Simmers et al., 2011), optic neuritis (Barton and Rizzo, 1994; Raz et al., 2011), injury to the cerebellum (Ivry and Diener, 1991; Nawrot and Rizzo, 1995), increase in light and movement thresholds as a non-specific sign of acquired brain injury (Mark and Pasamanick, 1958), and in association with various other visual and cognitive disorders after periventricular white matter damage (e.g., Weinstein et al., 2012), or in posterior variants of Alzheimer disease, accompanied with mental deterioration (Tsai and Mendez, 2009).

However, there were a few earlier case studies with acquired brain injury which provided evidence for a special brain structure underlying visual movement perception. Pötzl and Redlich (1911) reported a patient with bilateral occipital injury who was unable to perceive the movement of visual stimuli. The patient described her visual impression of moving objects as appearing at different successive positions. In contrast to this visual difficulty, she had normal color and form vision. However, because she also suffered from a severe visual field restriction, her impairment could perhaps be explained as an inability to maintain continuous fixation on a moving target as its visibility fluctuated when it moved within or outside the spared visual field. Goldstein and Gelb (1918) described a patient who had suffered an occipital gunshot wound. His visual field was also concentrically restricted beyond 30° eccentricity; his visual acuity, color vision and form discrimination were normal. In contrast, the patient had no impression of movement when confronted with moving visual objects but retained normal impression of movement with tactile stimulation. He stated that he could see visually presented objects at different positions, but never in motion between the positions. This experience did not depend on whether he maintained fixation on, or tracked, the moving target. In addition, he reported no perception of apparent movement. Similarly, one of Bodamer's prosopagnosic patients, HA, who had also lost movement vision, reported only successive changes in object position but had no impression of movement. Because the patient showed severe homonymous visual field restriction, and his visual acuity was only 0.60, part of his movement visual disorder may also be accounted for as a result of these adjunct visual deficits (Bodamer, 1947).

A complementary argument for acknowledging movement vision as a “special visual perception” had been put forward by Riddoch (1917a, p. 15). He performed careful examinations of the visual field in a group of people with posterior brain injury after gunshot wounds. He found preservation of movement vision, but loss of form and color vision, in homonymous parts of the visual field contralateral to the brain injury in nine out of ten patients. From his observations Riddoch (1917a) concluded, “that movement [vision] should be given a place among the stimuli which are recognized as originating visual perceptions” (p. 56). It should be mentioned here, that Riddoch did not use standardized methods to assess movement vision in his subjects, but “oscillated” the stimulus to determine the fields of movement vision; the response criterion was being “immediately conscious of ‘something’ moving” (p. 16). In this manner, Riddoch demonstrated that the scotomatous field was frequently smaller for moving stimuli than their static counterparts. Some authors have used Riddoch's observation on this so-called statokinetic dissociation, in which a stimulus is perceived during movement but not with static presentation, as an argument for the selective preservation and representation of visual motion perception (e.g., Vaina, 1989), others as evidence for a type of “blindsight” (for a detailed discussion, see Kentridge and Heywood, 1999). However, the selective sparing of movement vision is insufficient to draw conclusions about the functional segregation of visual cortex since the neural basis of such sparing remains unclear. The processing of movement stimuli is not confined to cortical mechanisms, in particular if direction and speed are not crucial parameters (Schiller and Stryker, 1974; Krauzlis, 2004). Moreover, as Zeki (1991) has pointed out, Riddoch presented positive evidence, i.e., loss of form and color vision, but preservation of movement vision, but did not provide essential evidence of the converse, namely the loss of movement vision with preservation of color and form vision. Furthermore, relative preservation of movement vision may result merely from the higher saliency of moving (and flickering), compared with stationary, stimuli which would have a higher probability of detection (for a review, see Treue, 2003). Reports of statokinetic dissociations are not uncommon. Homonymous visual field regions with depressed light sensitivity, impaired or even lost color and form vision, but spared detection of moving visual stimuli have been often reported after occipital damage and are known as cerebral amblyopia (e.g., Poppelreuter, 1917/1990; Teuber et al., 1960; Schiller et al., 2006; Zihl, 2011). Furthermore, statokinetic dissociations have been reported in cases with compression of the optic nerve and optic tract (Zappia et al., 1971), in retinal pathologies (Safran and Glaser, 1980; Gandolfo, 1996), and it may even be provoked in normal visual fields (Hudson and Wild, 1992; Schiller et al., 2006). Thus, preservation of (conscious) vision of moving stimuli but impaired detection of static stimuli is not just observed after injury to the striate cortex. But none of this implies that Riddoch (1917a,b) had not reliably assessed movement vision as a (conscious) visual quality in his patients. That conscious movement vision is possible without striate cortex has been convincingly demonstrated by Zeki and ffytche (1998) in a single case suffering from visual field loss since early childhood.

Poppelreuter (1917/1990) used dissociation of function in his studies on visual disturbances after occipital damage, as did Riddoch (1917a,b), to infer from his observations the genuine character of movement vision in the functional organization of the visual brain. This methodological approach, with some qualification (Dunn and Kirsner, 2003), has proved fruitful in elucidating the selective character of perceptual and cognitive functions (Teuber, 1955; Jones, 1983). Nevertheless, Riddoch's observations were not widely accepted despite their publication in prominent scientific journals and were neglected or dismissed by eminent authorities in the same field, for example, Holmes (1918, 1945) and Teuber (1960). The same was true of the few contemporaneous reports of disorders of movement vision in the German neurological and psychological scientific communities (Pötzl and Redlich, 1911; Goldstein and Gelb, 1918). The influence of such early work lay dormant for a number of years but interest in the neural basis of movement vision and its particular role in the brain organization of visual perception were reignited with the development of new methods to study the morphological and neurophysiological characteristics of the visual brain. Robust evidence for the concept of functional specialization in the visual cortex soon aroused (implicitly or explicitly) the expectation of a condition, which could result in a selective and specific loss of movement vision, an observation that could be taken as direct evidence for the existence of a genuine “motion system” in the visual brain. Such unequivocal evidence may be expected after experimental lesions to the cortical structure in question in monkeys, and after an acquired lesion in the respective cortical region in humans. It appears that nature was faster. The first reports on the effect of local experimental lesions in monkeys causing selective visual motion deficits appeared some years after the report of patient LM (Newsome et al., 1986, 1988; Newsome and Paré, 1988).

## The case of LM

### A brief history

On a Tuesday at the beginning of May 1980 a neurologist in Munich contacted JZ because of a 43-year-old female patient presenting with complaints about an “unusual, if not bizarre visual disorder mimicking agoraphobia.” She insisted of being unable to see motion and experienced the world as “restless,” with people changing their position so suddenly and unexpectedly that she loses them despite all efforts to keep them in sight. She had great difficulties with crossing roads; shopping was almost impossible during the day when many people were in the supermarket. She always needed much time to find out “what is going on.” The neurologist completed his report by adding that the patient had suffered a bilateral brain hemorrhage in October 1978 and had spent several months in a neurological rehabilitation center, unfortunately without significant remediation with respect to the visual disorder. Although he assumed that the visual disorder in question may have been caused by the hemorrhage in the posterior brain, he could not exclude a psychogenic component because he has never heard about such a strange visual disorder. JZ gave him a date for the neuropsychological assessment of the patient for the following week. A rather shy lady presented herself at the Max Planck Institute of Psychiatry. When asked for her major problems, she reported that since her brain hemorrhage she could no longer see movements. “People, dogs, and cars appear restless, are suddenly here and then there, but disappear in between. Very often I don't even know where they have left, because they move too fast, so I lose them quite often.” Fluids appeared frozen, like a glacier, which caused great difficulty, for example, with pouring tea or coffee into a cup; filling a glass with water became impossible. Most events were much too fast for her and she needed a considerable time to perform even simple routine activities, such as cutting bread or using the vacuum cleaner. She could no longer use the tube, bus or tram, which severely restricted her mobility. She also found it very irritating to meet friends and have a chat with them because she could not respond in time to their handshake and because she found their moving hand disturbing. In addition, the experience of talking to them was very unpleasant because she had to avoid watching their (changing) facial expressions while speaking, in particular, their lips seem to “jump rapidly up and down, and I am very often unable to listen to what they were saying.” In contrast, when people, faces, objects and cars were stationary, she had no difficulty in seeing them “clearly” and could recognize them immediately and accurately. The perception of colors had not changed, and she reported no difficulty with perceiving the position of objects and judging correctly both how far away they were and the distances between them. She reported that reading took more time than before, writing had become somehow difficult. Psychiatric examination revealed no psychopathological symptoms, in particular depression, anxiety or agoraphobia.

LM's own detailed report of her visual difficulties indicated that her visual disorder was probably both specific and selective. She was fully aware of her visual disorder and its consequences in everyday life activities, which she correctly attributed to the brain injury she had suffered, without any sign of anosognosia. Her description of motion blindness resembled closely that of the patients reported by Pötzl and Redlich (1911) and Goldstein and Gelb (1918).

**Figure 1:**
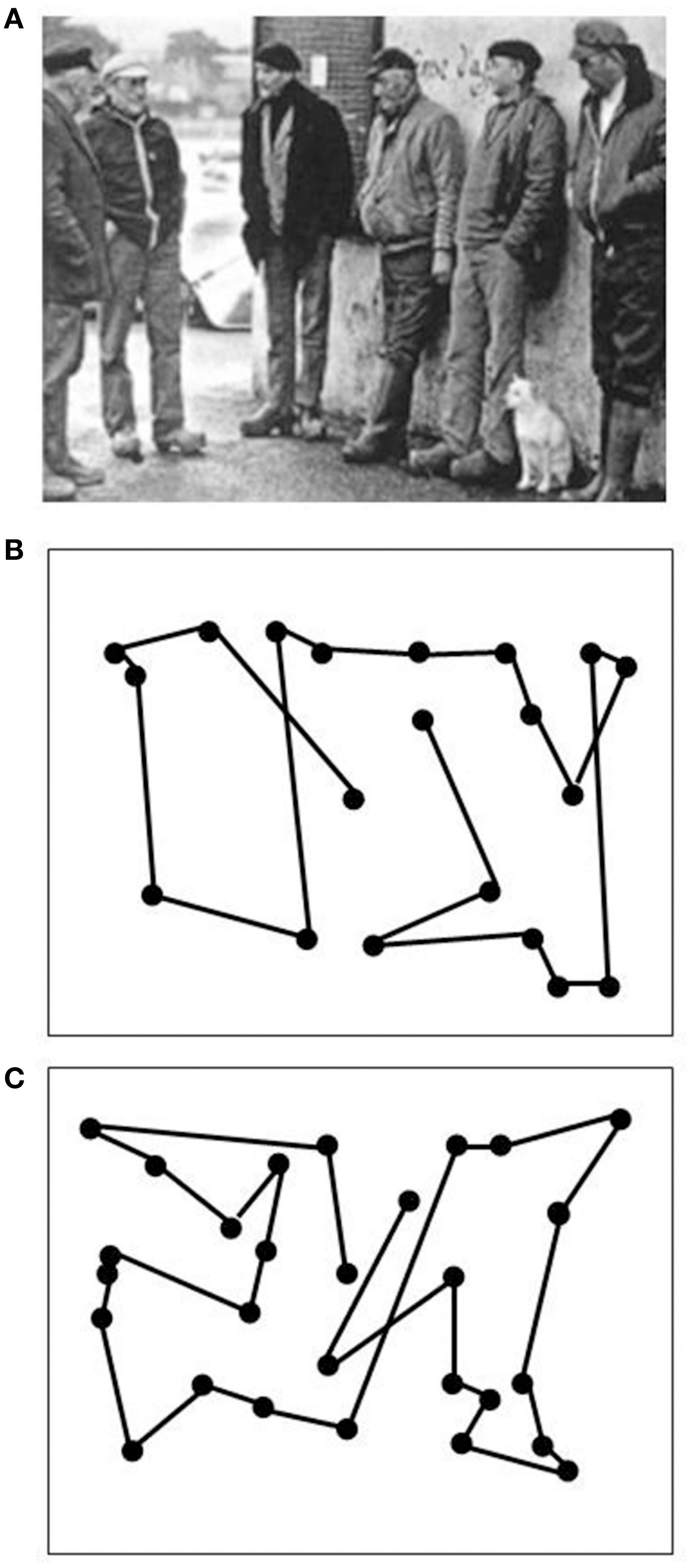
**Oculomotor scanning patterns during the inspection of a scene (A) in an age-matched normal subject (B) and in LM (C)**. Dots indicate fixation positions, lines saccadic eye shifts. Both subjects reported all relevant items. Scanning time was 13.6 s for the normal subject and 26.6 s for LM. Note similar correspondence of scanning patterns to the spatial configuration of the scene in both subjects.

**Figure 2:**
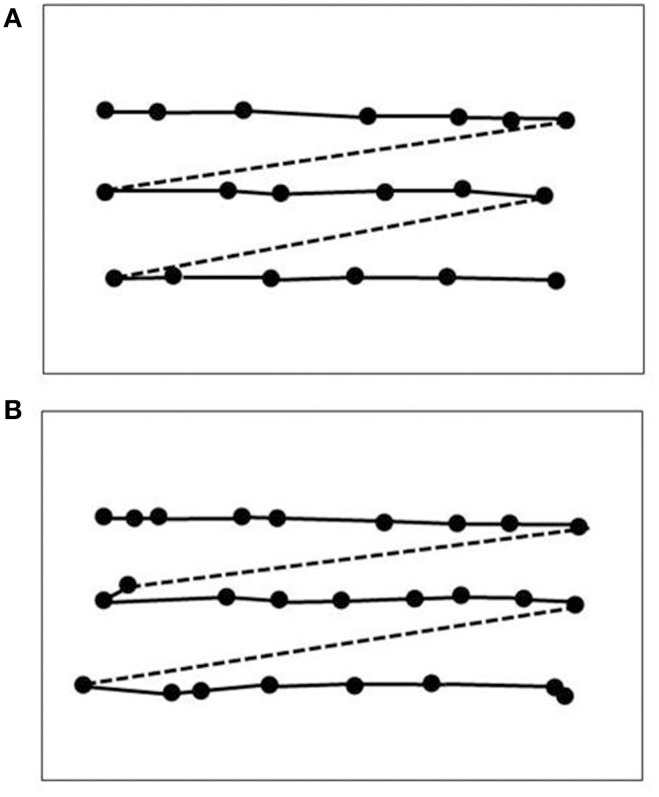
**Reading eye movement patterns in an age-matched normal subject (A; same as in Figure 1B) and in LM (B)**. Dots indicate fixation positions. Reading performance in the normal subject was 156 words per minute (wpm), in LM 72 words per minute. The slowness in LM can be explained by a higher number of fixation repetitions (22.7% in LM vs. 4.3% in the normal subject) and in longer fixation durations (0.31 s on average in LM vs. 0.22 s in the normal subject).

**Figure 3:**
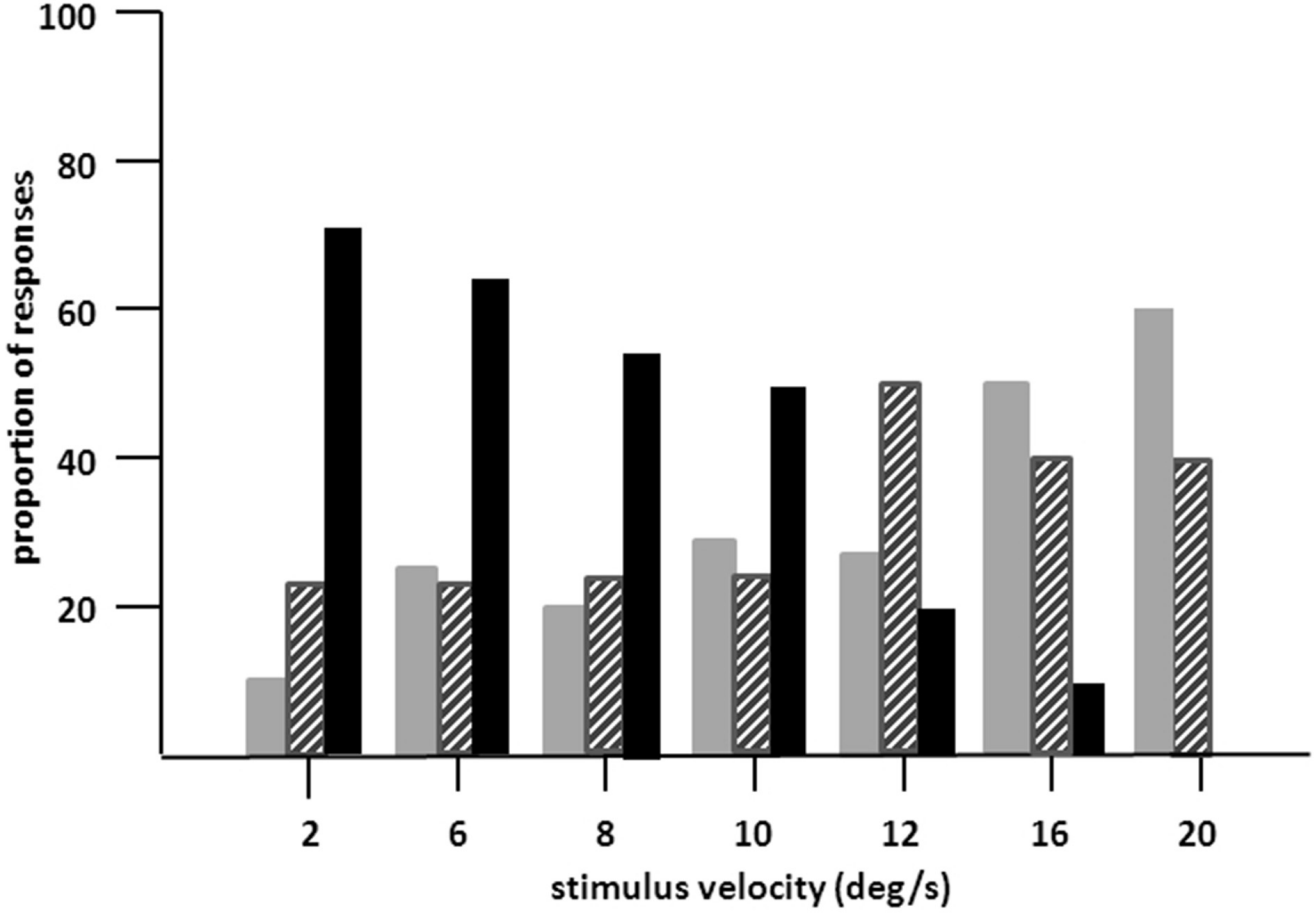
**Proportion of “no” (gray bars), “uncertain” (hatched bars), and “yes” responses (dark bars) of LM in 20 trials in stimulus velocities ranging from 2°/s to 20°/s**. Moving path length was 20°. LM's task was to indicate verbally, whether she can see the stimulus in motion (yes responses), was not sure about motion (uncertain responses) or could not see motion at all (no responses; 10 trials per velocity). Presentation time was unlimited, but was usually between 2 and 5 s. Note increase in “no” and decrease in “yes” responses with increasing velocities.

**Figure 4:**
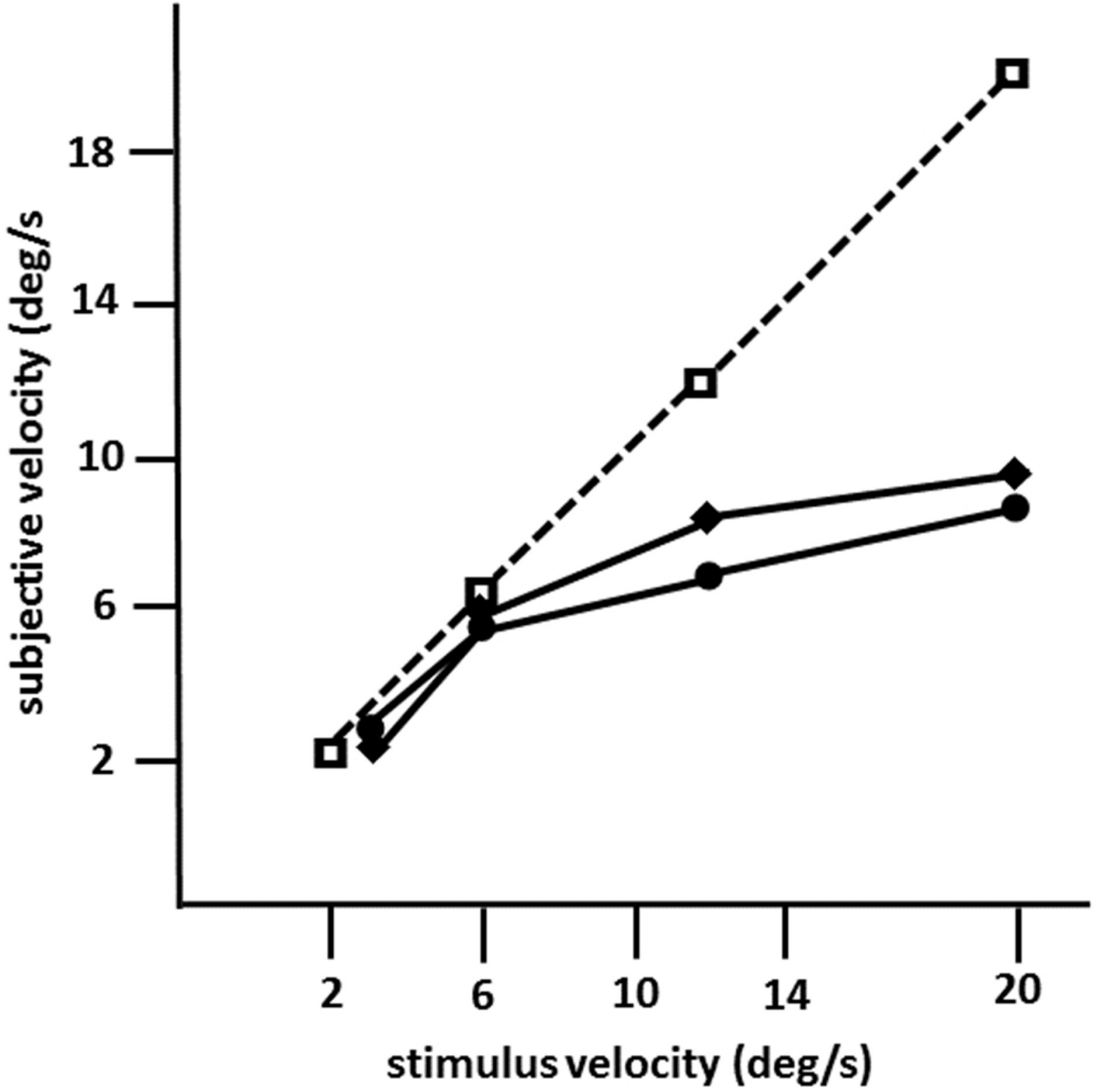
**Mean subjective velocities for LM in 1982 (circles) and in 1990 (diamonds), and in an age-matched normal subject (squares; same as in Figures 1B, 2A; 10 trials per velocity) as a function of stimulus velocity calculated from motion prediction responses (modified after Zihl et al., 1983, 1991)**. LM was instructed to press a key to start the target in motion, and to press it again when she judged that the now invisible target had reached a red marker behind a mask. The path of horizontal movement was 10°; the length of the path behind a mask was 20°. Note that motion prediction accuracy in LM dropped for stimulus velocities >6°/s on both occasions.

Data on LM's visual capacities and movement vision profiles have been reported in detail elsewhere (Zihl et al., 1983, 1991; Zeki, 1991; Rizzo et al., 1995; Heywood and Zihl, 1999). We will focus here on two aspects of the significance of the case of LM: the specificity and the selectivity of her visual disorder, and compare them with other cases with impaired motion vision, before and after 1983. Specifity means that LM's motion blindness is not the result of other visual or non-visual disorders, which could putatively explain her severe impairment in detecting moving visual stimuli and discriminating their directions and velocities. Visual fields for detection of light, critical flicker fusion (CFF) and detection of simultaneously presented stimuli in both hemifields, color and form recognition, visual acuity and contrast sensitivity, temporal separation and temporal order of visual stimuli, visual localisation and stereopsis, and visual recognition were all normal on formal testing. Furthermore, attention (apart from non-specific cognitive slowing; see below), visual and verbal memory and cognitive flexibility were not impaired; in particular, there were no signs of perseveration. In addition, LM showed no oculomotor or hand motor dysfunctions that could interfere with visually guided eye- and hand-movements. Her eye movement patterns during inspection of a scene and in reading were normal (Figures 1, 2). LM had no difficulties with understanding verbal instructions and keeping them in mind during testing sessions, with responding to stimuli verbally or with hand motor responses, switching between stimulus and response categories, with commenting on her responses and reporting lucidly her visual impressions despite mild anomic aphasia. Taken together, these facts support the notion that the motion blindness in LM cannot be explained by other dysfunctions, either visual or non-visual in nature, but represents a strikingly specific visual disorder. Selectivity of LM's motion blindness refers to the fact that her motion blindness was the only and exclusive deficit caused by her bilateral injury to the “visual brain.” Part of the evidence has already been described above. In addition, color vision, form and object vision, visual spatial functions, including visual localisation, distance and depth perception, object and face perception, visual recognition of objects, faces, letters and places, and reading and calculation were not impaired. Writing was not impaired, but slowed because of interference with vision of the motion of the pencil and hand. Similarly, visuo-constructive abilities were slowed, but LM did not exhibit any symptoms of ideomotor or ideational apraxia. In summary, motion blindness in LM presents as a highly selective visual disorder. Figure 3 shows the outcome of an experiment on movement vision performed in 1985. Table 1 summarizes the various components of vision and movement vision studied in LM and reported between 1983 and 2000 in 12 research papers and one book chapter, with 25 different authors involved. It becomes clear that the study of LM contains a comprehensive list of experimental conditions, including a follow-up study (Zihl et al., 1991) in which some of the experiments reported in the first paper (Zihl et al., 1983) were repeated with nearly identical outcomes (Figure 4). This fulfills an essential prerequisite for valid and reliable research, for which reproducibility represents a “cornerstone of science” (Simons, 2014, p. 76). In none of the earlier or later studies was movement vision tested in so many conditions to unequivocally establish specificity and selectivity of visual motion dysfunction or visual motion blindness. However, these other studies have added further evidence about various aspects of visual motion perception (see Table 2, for a summary), for example, dissociation of 3D-structure from motion and stereopsis (Vaina, 1989); impairment in visual motion perception in the hemifield contralateral to unilateral posterior brain injury (Plant and Nakayama, 1993; Plant et al., 1993; Greenlee and Smith, 1997; Schenk and Zihl, 1997a,b; Braun et al., 1998); direction-selective visual motion impairment (Blanke et al., 2003a); dissociation of processing of various types of visual motion stimuli (Billino et al., 2009; Vaina et al., 2010), and the transient condition of visual motion blindness (Cooper et al., 2012). Although there is no reason to believe that the reported visual motion impairments in patients with uni- or bilateral posterior brain injury are non-specific, it is not immoderate to remark that none of the other patients reported in the literature has been documented in such detail with respect to the specificity and selectivity of the disorder as LM. Of course, selectivity cannot be expected in each case because it depends on the extent of brain injury. However, specificity should be demonstrated in each case, otherwise impaired movement vision may, at least in part, be caused and thus explained by other visual and/or by non-visual deficits and would then not represent a genuine visual deficit.

**Table 1:** **Summary of outcomes of visual (A) and movement vision (B) assessment in LM**.

**(A) VISUAL FUNCTIONS AND CAPACITIES**		
Visual fields	+	Zihl et al., 1983
Visual acuity (far and near)	+	Zihl et al., 1983
Spatial contrast sensitivity	(+)	Hess et al., 1989
Temporal contrast sensitivity	(+)	Hess et al., 1989
Critical flicker fusion	+	Zihl et al., 1983
Temporal separation	+	Zihl et al., 1983
Color discrimination	+	Zihl et al., 1983
Stereopsis	(+)	Zihl et al., 1983; Rizzo et al., 1995
Visual reaction time	(**−**)	Zihl et al., 1983
DSS detection	+	Zihl et al., 1983
Visual localization	+	Zihl et al., 1983
Visual form discrimination	+	Zihl et al., 1983; Rizzo et al., 1995
2−D and 3−D shape perception	+	Rizzo et al., 1995
Visual recognition	+	Zihl et al., 1983
**(B) MOVEMENT VISION**		
Movement detection in the foveal visual field	**−**	Zihl et al., 1983
Movement detection in the peripheral visual field	**−**	Zihl et al., 1983
Discrimination of movement direction	(**−**)	Zihl et al., 1983; Shipp et al., 1994; McLeod et al., 1996; Marcar et al., 1997
Movement vision, >6°/s	**−**	Zihl et al., 1983, 1991
Motion prediction (horizontal direction), >6°/s	**−**	Zihl et al., 1983, 1991
Coherence of visual motion perception, >6°/s	**−**	Baker et al., 1991
Visual search for moving stimulus	**−**	McLeod et al., 1989
2−D and 3−D shape and structure from motion	(**−**)	Rizzo et al., 1995
Apparent motion	(**−**)	Zihl et al., 1983; Hess et al., 1989
Motion aftereffects	**−**	Zihl et al., 1983
Biological motion perception	(**−**)	McLeod et al., 1996

*+, normal; (+), mild impairment; (−), moderate impairment, − loss; DSS detection: detection of stimuli in a double simultaneous stimulation condition. References are in [brackets]*.

**Table 2:** **Synopsis of cases reported with motion blindness or impaired movement vision (1911–2014)**.

**Author(s)/year**	***n***	**BI**	**VF**	**Acuity**	**CS**	**stereo**	**Vis loc**	**Movement vision**
Pötzl and Redlich, 1911	1	bil	bil	NR	NR	NR	NR	Subjective report
Goldstein and Gelb, 1918	1	bil	bil	+	NR	NR	NR	Subjective report
Bodamer, 1947	1	bil	bil	0.50	NR	NR	NR	Subjective report
Vaina, 1989	18	uni	NR	+	NA	8/18−	NR	Velocity comparison and SFM impaired
Plant et al., 1993	11	uni	7	+	+	NR	NR	Elevated thresholds for motion direction in the CL hemifield
Greenlee et al., 1995	23	uni	2	NR	NR	NR	NR	Threshold elevation for velocity discrimination
Greenlee and Smith, 1997^*^	21	uni	3	NR	NR	NR	NR	Threshold elevation for direction of motion and speed discrimination
Schenk and Zihl, 1997a	32	uni	5	+	NR	NR	NR	Impaired CM perception
Schenk and Zihl, 1997b^**^	39	37/2^**^	7	+	NR	NR	NR	Impaired form-from-motion perception
Braun et al., 1998	9	uni	2	+	NR	NR	NR	Threshold elevation for CM in the CL hemifield
Billino et al., 2009	23	uni	4	+	+	+	NR	Impaired perception in translational motion (*n* = 3)
Blanke et al., 2003a	11	uni	7 with VFD	NR	NR	NR	NR	Impaired discrimination of motion direction
Vaina et al., 2010	57	uni	NR	NR	NR	25/572**–**	+	Impaired movement vision of different type in 77% of cases

*NR, not reported/assessed; n, number of cases; BI, brain injury, uni, bil, uni-and bilateral brain injury, respectively; VFD, visual field loss (uni: unilateral, bil: bilateral visual field loss, respectively); CS, contrast sensitivity; Stereo, Stereopsis; Vis loc, visual localisation; Mov vision, movement vision. +, normal; −, impaired/lost. SFM, Structure-from-motion; CM, coherent motion; CL, contralateral. Visual acuity refers to decimal near acuity (1.0 ~ 100%)*.

* *10 subjects were also included in Greenlee et al. (1995)*.

** *31 subjects were also included in Schenk and Zihl (1997a); 2 subjects exhibited bilateral posterior brain injury. 8/18- and 25/57-: 8 out of 18 cases and 25 out of 57 cases impaired*.

Interestingly, experimental data from normal observers in various movement vision tasks underline the particular character of LM's specific visual disorder. For example, Kennedy et al. (1972) reported very precise visual velocity estimation in the range of 0.8°/s to 11°/s. High accuracy in visual motion prediction in normal subjects was reported by Wiener (1962) and Rosenbaum (1975); LM showed, in contrast, severe impairment in both tasks. Sekuler and Ball (1977) found that the predictability of movement direction improved performance in normal subjects by about 20%. LM did not benefit either from predictability or from feedback. Clatworthy and Frisby (1973) investigated the effect of adaptation to real movement on the perception of subsequent apparent movement and found a marked carry-over effect of adaptation. LM experienced no phi-movement, except in the short-range (Hess et al., 1989), suggesting that the same movement-detecting mechanism mediates both real and apparent movement phenomena (Gregory and Harris, 1984; Newsome et al., 1986). Evidence for this derives from neuroimaging participants while they viewed stimuli in apparent motion. By adjusting the spatial and temporal properties of spatially alternating stimuli, it is possible to produce displays which are ambiguous, i.e., perception alternates between a single stimulus in motion or two stationary blinking stimuli. Early visual areas responded equally under the two conditions, however a region including area V5/MT was activated during the perception of apparent motion (Muckli et al., 2002). Finally, Anstis and Ito (2010) have shown that smooth pursuit eye-movements are guided by real stimuli and not by retinal signals. Therefore, LM's difficulty with visually-guided smooth pursuit eye-movements is more likely of central origin, caused by her cerebral motion blindness, and not by dysfunction of her peripheral visual system.

## Is there a visual motion “center” or module in the brain?

**Figure 5:**
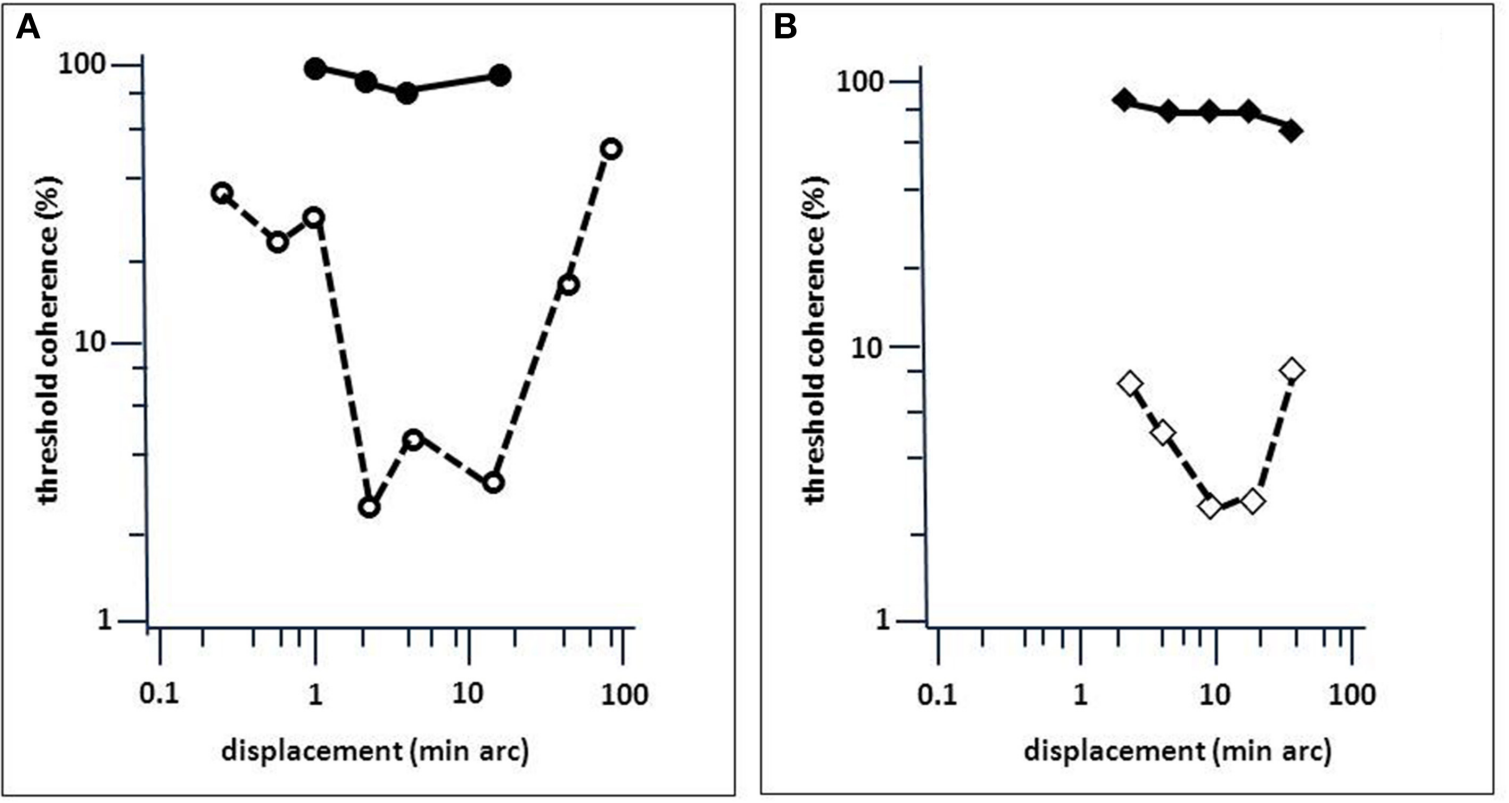
**Comparison of LM's performance in a motion-coherence task with that of MT-lesioned monkey (Newsome and Paré, 1988)**. **(A)** Threshold coherence values for the Movshon noise stimulus as a function of spatial stimulus displacement for LM (filled circles) and an age-matched normal subject (open circles; same as in Figure 4). **(B)** Same as **(A)**, but for monkeys before (open diamonds) and after acute MT lesion (filled diamonds). Presentation time was 1 s. The subjects were required to indicate the perceived (or guessed) direction of stimulus motion (left or right). Note the similarity in the effect of brain injury to motion coherence perception (modified after Baker et al., 1991).

LM's bilateral brain injury was caused by thrombosis of cortical veins in cerebral sinovenous occlusion and affected the middle and superior temporal gyri, extending into the lateral occipital gyri. Thus, the bilaterally symmetric brain injury, which was more extensive on the left side, was mainly located in the lateral occipital cortex and the underlying white matter with the main focus in the upper (cranial) banks of the anterior occipital sulcus (Zihl et al., 1983, 1991). This is consistent with the location of area V5/MT and its surroundings in primates and humans, which occupy the temporo-parieto-occipital pit at the boundaries of Brodmann areas 19 and 37 (for a review, see Zeki, 1991). Later studies have confirmed that the principal location of brain injury causing impaired visual motion perception is in the region bordering lateral occipital and superior temporal cortex (Plant and Nakayama, 1993; Greenlee et al., 1995; Greenlee and Smith, 1997). However, as Blanke et al. (2003a) have shown, injury to the posterior parietal cortex may also cause dysfunction of motion vision. Similar observations have been reported by Vaina et al. (2010), who compared behavioral and morphological MRI-data in 57 patients with visual motion impairments after stroke. Differences in task performance, including direction and speed discrimination, radial and non-radial motion coherence detection, and motion discontinuity detection were correlated with injury localisation. Occipito-temporal and (pre-) frontal injury was not associated with impaired task performance, but occipito-parietal injury (areas VIP, AIP, LIP, and MIP) was associated with substantial impairments. The fact, that cortical areas other than V5/MT are essentially involved in visual motion processing is not a compelling argument against the idea of a single structure in the extrastriate visual cortex, which is crucial for the processing of visual motion signals and thus for visual motion perception. In addition, interactions between motion processing units should also be considered, i.e., with respect to white matter injury (Nishida, 2011; see also discussions in Zihl et al., 1983; Zeki, 1991). Considering the combined evidence it appears, however, that V5/MT is the most probable candidate as the “visual motion center.” Neurophysiological and neurobehavioral data from primates (e.g., Movshon and Newsome, 1992) and brain imaging (e.g., Watson et al., 1993; Aspell et al., 2005), as well as stimulation data from humans (e.g., Beckers and Zeki, 1995; Becker et al., 2013), are consistent with this view. Further evidence comes from a study by Marcar et al. (1997) who compared LM's motion blindness with that of macaque monkeys with area MT removed. They found a close correspondence between patterns of impairments indicating that LM's loss of movement vision is attributable to total loss of, or extensive damage to, a cortical visual area that is the human equivalent of area MT and perhaps its adjacent areas. In addition, LM showed a similar deficit in a motion coherence task (Baker et al., 1991) to monkeys with bilateral MT ablation (Newsome and Paré, 1988; Figure 5). Moreover, Britten et al. (1992) and Celebrini and Newsome (1994) have convincingly shown that psychophysical data and neuronal responses in monkey MT show high correspondence in a direction discrimination task; sensitivity in these neurons was very similar to the psychophysical sensitivity at the behavioral level. Thus, the combined evidence strongly supports the idea of at least regional specialization for movement vision in extrastriate visual cortex (Vaina et al., 2005), with an additional role of the cerebellum (Ivry and Diener, 1991; Nawrot and Rizzo, 1995), which is poorly understood.

## Behavioral consequences of visual motion blindness

**Figure 6:**
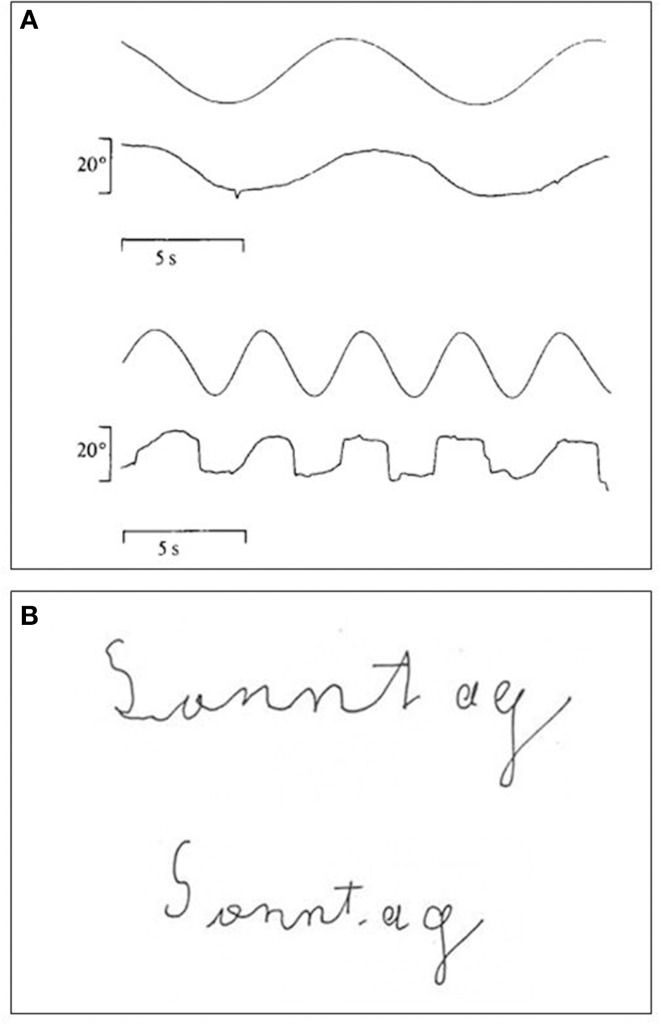
**(A)** Recordings of LM's smooth pursuit eye movements to a target moving either at 4°/s (upper trace) or at 8°/s (lower trace). Note deterioration of smooth pursuit at the higher velocity (modified after Zihl et al., 1983; © Oxford University Press with permission). **(B)** Handwriting with eyes open (upper writing) and eyes closed (lower writing). Time taken for writing was 4 s with eyes closed and 26 s with eyes open (modified after Heywood and Zihl, 1999, © Psychology Press with permission). Note better writing with eyes closed.

As mentioned earlier, LM was referred by her neurologist because of a visual disorder, most likely caused by brain injury and a behavioral disorder, which presented itself as an aversion to crowded places. The neurologist did not assume any association between the two disorders. In fact, her motion blindness caused severe impairments in all activities that are either guided by, or are associated with, movement vision. Pursuit eye-movements were only possible for slowly moving stimuli (Zihl et al., 1983; Figure 6A). Reaching for and grasping of moving objects was difficult, as was manipulating objects with her hands moving. Walking was difficult because LM could not watch her moving feet without being irritated; in addition, she was distracted (if not captured) in an uncomfortable way by people approaching or overtaking her. As a consequence, she used to stop walking and waited until people were out of sight. Interestingly, in normal observers self-motion, such as walking, apparently subtracts perceived visual speed (Durgin et al., 2005), which should have supported LM's coping with moving signals, but it did not. Furthermore, she had difficulties keeping her body in balance because of interference of visuo-vestibular interactions with visual stimulus movement (Paulus and Zihl, 1989). When only a single person approached her, she could detect the “restless” person, but could not tell a person's direction of movement, consistent with impaired motion-in-depth perception (Zihl et al., 1983; Rizzo et al., 1995). The presence of additional stationary people, perhaps providing figure-ground segregation, was not helpful which is consistent with the observation that adding static noise to a moving stimulus severely affected her movement direction judgments (Shipp et al., 1994; McLeod et al., 1996). These difficulties caused a severe visual handicap in all activities-of-daily-living including personal hygiene, cooking, cleaning, shopping, using public transport, and meeting friends. Nevertheless, LM learned to cope successfully with these adverse conditions by daily systematic practice under supervision over several months. For example, she learned to overcome her difficulties with pouring water, tea, coffee or milk in a cup or glass, by using her intact distance perception to stop pouring fluids when they reached about 1 cm below the rim. She had difficulties slicing bread, because of the movement of the knife, but learned to put the knife in the appropriate position and then just make the cut without observing the knife. She chose to shop when the supermarket was nearly empty but never used a trolley; when somebody else appeared in her field of view, she stopped and waited until the person had passed her. She learned to use again public transport by avoiding watching people entering alighting and following the last passenger in front of her while looking only at his or her back, i.e., the body part that was least “restless.” When she was eventually in the compartment of the tube, bus or tram, she searched for a handhold and kept her fixation at a given position until exit. She got to know new friends and met with them regularly for various outdoor activities. She informed them in advance that she is unable to look at their face when they are speaking because otherwise she has difficulty in listening to what they are saying. This is consistent with the interference she experienced between hearing and facial and especially lip movements (see Campbell et al., 1997). As with self-motion, moving faces did not make facial recognition easier for LM, as in normal observers (Lander and Chuang, 2005), but more difficult. The development of successful coping strategies to compensate for her inability to process visual motion stimuli is in sharp contrast to the chronic nature of her motion blindness, which was found essentially unchanged when examined 8 years after the first report (Zihl et al., 1991). Coping strategies consisted of a mixture of active adaptation and avoidance behavior. Avoiding watching moving stimuli had a positive effect on guiding finger- and hand- movements (Zihl et al., 1983) and on writing (Heywood and Zihl, 1999; Figure 6B). Although LM became less anxious in public over the years she still avoided crowded places unless she was in company, when she sought reassurance by linking arms while walking. Sometimes she was, however, still frightened, for example, when people, dogs or cars suddenly “appeared or disappeared” in front of her. However, she never showed phobia in the psychiatric sense of the term, as did a patient reported by Blanke et al. (2003b), but took her unusual and often uncomfortable visual experiences with great patience and humor.

## Conclusions and final comments

The main outcome of the comprehensive assessment of LM, including neuropsychological examination, testing of visual functions and capacities, and, in particular, movement vision, revealed a highly specific and selective loss of visual motion perception and, consequently, impaired visually guided activities that depend crucially on the ability to process motion signals. The terms “motion” “blindness” and “cerebral akinetopsia” appear more than appropriate to denote this unusual visual disorder, even though LM possessed some kind of residual movement vision. The severity of LM's disability is underlined by the fact that she was even more impaired in moving stimulus conditions that are known to enhance perception and guidance of behavior in normal observers. Combined psychophysical, neuropsychological, neuroanatomical, neurophysiological and behavioral data after experimental lesions in primates support Riddoch's (1917a,b) and Gibson's notion (1954), that movement vision is a special visual perceptual quality. This assumption is further supported by developmental findings on the very early existence of visual motion sensitivity (e.g., Freedland and Dannemiller, 1987; Aslin and Shea, 1990; Armstrong et al., 2011; Mohring et al., 2012), indicating, that motion vision may possess an innate basis, and thus is an inherent capacity of the visual brain.

There is agreement that movement vision, like color vision, is subserved by an extrastriate cortical structure specialized for processing motion signals. It appears that this structure corresponds to visual area V5/MT, and its connected satellite regions. There is evidence of a constellation of visual areas involved in the processing of motion signals of a particular nature. This would explain dissociations of visual motion impairments and thus heterogeneous patterns of deficits in other patients with posterior brain injury. For example, motion signals can be carried by variations in luminance or color or carried by differences in contrast, texture and disparity (first- and second-order motion, respectively). The perception of such motion can be differentially affected by brain damage (Greenlee and Smith, 1997). Similarly, despite the severity of L. M.'s disorder, she is able to perceive some complex forms of motion normally. For example, when small lights are attached to the joints of an actor who performs actions while being filmed in the dark, the pattern of moving dots defines so-called biological motion such as walking, running and jumping (so-called Johansson figures). Despite the impoverished nature of the display and the small number of lights visible in the dark, their moving configuration provides compelling percepts of human actions. L. M. could readily identify such actions. Nevertheless, biological motion processing is not entirely normal. Having identified, for example, a walking figure, LM is unable to report its direction of motion or whether it is retreating or approaching. If a small number of stationary dots are added to the display, LM has difficulty in identifying the figure, presumably because of as difficulty in segregating the moving from stationary dots (McLeod et al., 1996).

LM's pattern of impairments is consistent with the view that motion perception is supported by multiple brain areas and is involved in a range of perceptual tasks. (Newsome and Paré, 1988; Rizzo et al., 1995; McLeod et al., 1996; Murray et al., 2003; Noguchi et al., 2005; Gilaie-Dotan et al., 2011). Motion areas are not confined to the dorsal processing stream. For example, biological motion involves a number of temporal, frontal and parietal cortical regions (Grossman and Blake, 2002; Puce and Perrett, 2003; Saygin et al., 2004; Blake and Shiffrar, 2007; Saygin, 2007; Rizzolatti and Sinigaglia, 2010) and, not only dissociates from other kinds of motion perception (e.g., Vaina et al., 1990; Battelli et al., 2003; Saygin, 2007), but can also dissociate from processing of visual form. Gilaie-Dotan et al. (2011) describe a case of developmental visual agnosia who, while impaired at extracting form from non-biological motion, retains the ability to use biological motion cues. The processing of biological motion can therefore dissociate from other form processing, even in the ventral pathway. The contribution of the ventral pathway to motion processing has also been shown in a study assessing the performance of five patients with left or right ventral lesions on a number of psychophysical tasks which assessed both non-form-based motion (e.g., detection and motion coherence) and form-based motion (e.g., structure-from-motion), at a wide range of motion speeds (Gilaie-Dotan et al., 2013). Right ventral lesions resulted in impairments in motion perception for both form- and, surprisingly, non-form-based motion at slow and fast speeds. This suggests that it is not only the dorsal visual pathway that is critical for motion perception. The authors propose that right ventral cortex is implicated in processing of motion in the central visual field. In contrast, MT/V5 is concerned with peripheral motion in the contralateral field. Patient LM showed greater preservation of movement perception up to 15° of eccentricity with more substantial impairments in the visual periphery, where motion processing was confined to discriminating moving and stationary targets (Zihl et al., 1983). Several studies have suggested that dorsal and ventral visual areas are involved with fast and slow motion, respectively (Gegenfurtner and Hawken, 1996; Burr and Thompson, 2011; Hayward et al., 2011; Narasimhan and Giaschi, 2012). The integrity of the ventral stream in LM may therefore account for her relatively better performance at detecting low speeds.

Although it seems still an open issue as to whether V5/MT is the crucial structure for movement vision, it appears that in LM this structure and the majority of other structures involved in the processing of other kinds of visual motion signals have been destroyed by the bilateral symmetrical brain injury she suffered (see also Marcar et al., 1997). The extent of the injury, encroaching on the cluster of brain areas concerned with motion processing, may also explain why LM, unlike non-human primates where the ablation is largely restricted to V5/MT, did not show any recovery of movement vision at all despite intensive practice with coping strategies.

Of course, selectivity of a functional deficit in humans depends heavily on the size of the associated brain injury. Such selectivity will be the exception, not the rule, since brain injury is usually larger than the size of the cortical structure in question (Campbell, 1905). In this respect the selectivity of motion blindness in LM was clearly such an exception; one would have predicted many more functional deficits than were found. The evidence for selectivity does, however, not come from the anatomical analysis of LM's brain injury, but from a very comprehensive behavioral assessment of her visual and non-visual functions and abilities. This detailed assessment was, in addition, the fundamental basis for the proof of specificity of motion blindness in LM. It seems reasonable, but also important from a methodological point of view, to consider at least a critical minimum of assessment of visual and cognitive function in patients with impaired motion vision to guarantee an adequate degree of specificity.

In conclusion, LM has made a very significant contribution to our understanding of visual movement perception and the underlying brain functions and structures. Because of the selectivity and specificity of her motion blindness, she represents undoubtedly a “key” case in the neuroscience of vision. In this sense, her case was indeed a moving story: on the one hand she moved research on movement vision, on the other her story moved everybody who participated in the many experiments on which she has collaborated with great enthusiasm. For this engagement, we express our deep respect and our gratitude to her. LM fell in a comatose state for 2 weeks as a result of a second brain hemorrhage, and died on the 20th of January, 2003.

### Conflict of interest statement

The authors declare that the research was conducted in the absence of any commercial or financial relationships that could be construed as a potential conflict of interest.
